# What does Kombucha Tea Improve the Stool Passage in Patients with Schizophrenia? A Preliminary Results of A Randomized, Double-blind Placebo-controlled Clinical Study

**DOI:** 10.1192/j.eurpsy.2025.2221

**Published:** 2025-08-26

**Authors:** A.-Y. Hong, Y.-J. Shih, Y.-J. Yang

**Affiliations:** 1 Department of General Psychiatry; 2 Department of Nursing, Tsao-Tun Psychiatric Center, Nantou county; 3 Department of Healthcare Administration, Asia University, Taichung, Taiwan, Province of China

## Abstract

**Introduction:**

Patients with schizophrenia frequently have difficulties in defecation which may lead to adverse health consequences. Many interventions have been proposed to resolve the problems but not usually effective. Kombucha tea has been advocated for their effects on gut microbiota and thought to improve the stool passage in healthy population. However, the relevant evidence was insufficient in patients with schizophrenia. In this study, the research team tried to evaluate the effectiveness of Kombucha tea in the clinical settings.

**Objectives:**

This study aimed to evaluate both subjective and objective amelioration of stool passage in constipated patients with schizophrenia.

**Methods:**

Schizophrenic inpatients who took laxative medications or had subjective difficulties in stool passage were eligible for the study. The protocol was approved by the IRB of Tsao-Tun Psychiatric Center and registered on the trial registry of Clinicaltrial.gov. After obtaining consents and initial screening, the recruited participants were randomly allocated into either the control or intervention groups. Participants in the control group were provided with flavored lemon tea while those in the intervention group drank commercial Kombucha tea. Every morning during 8-week period, participants in both groups were provided with the drinks bottled in opaque plastic bottles filled with the same volume (200ml) and similar taste.

The multidisciplinary team collected the demographic profiles, clinical details, kinesiological data, cognitive condition (measured with the MoCA) and psychiatric status (measured with the BPRS). The outcome data were collected through interview and medical record review by the researchers independent from allocation of the groups, and were computed with the statistical software JASP with the statistical significance at the 5% level (p<0.05).

**Results:**

79 schizophrenic patients were enrolled in the study, and the key data were illustrated at the attached Table. All the variables at the baseline showed no statistical significance between groups. In terms of the outcomes, the means of objective measurement of stool passage showed no statistical significance between groups. In contrast, the subjective measurement showed the ease of defecation difficulties, trending towards ‘smoother and easier defecation’ (p<0.05).

**Image 1:**

**Figure:**
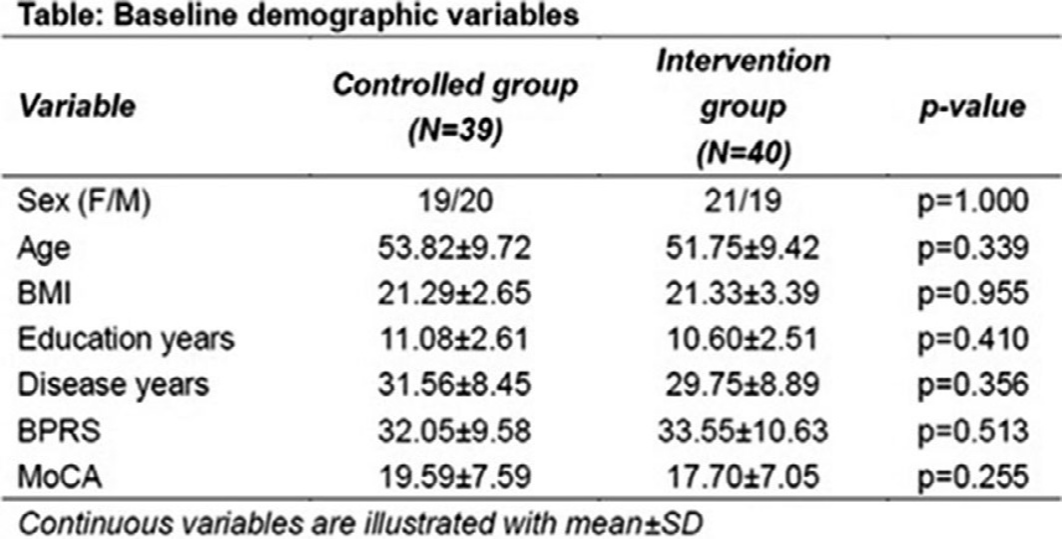

**Conclusions:**

Our preliminary analyses indicated that Kombucha tea improved the subjective outcome of stool passage in schizophrenic patients with constipations after taking Kombucha tea drinks while no statistical significance was observed in the objective measurements. To the best of our knowledge, this study is one of the very few studies exploring the effectiveness of Kombucha tea in ameliorating difficulties in stool passage in the patients with schizophrenia and it shed the light of future research.

**Disclosure of Interest:**

None Declared

